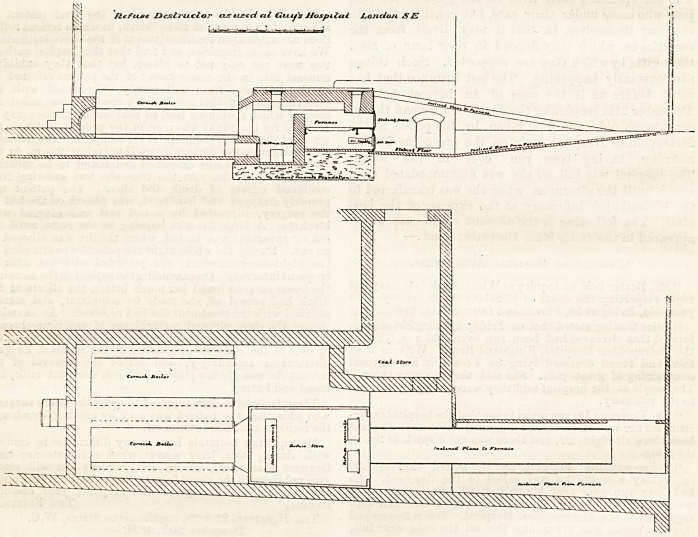# Practical Departments

**Published:** 1897-01-02

**Authors:** 


### PRACTICAL DEPARTMENTS.

### THE DESTRUCTION OF HOSPITAL REFUSE.

How to destroy the refuse produced in a hospital rapidly, completely, and without offence is an important problem in hospital management, and one which in many institutions has been treated more lightly than it deserves. The more we recognise that the first duty of a hospital, in order that it may do its best for the patients whom it receives, is to be clean, and the more clearly we understand that the worst form of dirt, from a surgical point of view, consists of soiled dressings, the more important does it appear that a hospital should be possessed of some means by which these may be effectually got rid of with the smallest possible delay.

Probably the worst way of dealing with such refuse is to let it be removed by the sanitary authorities as "dust." This always involves delay, frequently involves difficulties with the dustmen, and in other ways is open to many objections. A much better plan is to have it burned; the difficulty is where to burn it. In many hospitals all such matters are placed in the boiler furnace, and when they are small in quantity this is an effectual way of getting rid of them. In any large hospital, however, there are many things besides dressings that are better got rid of by fire, and directly any attempt is made to burn any large quantity of offensive material under a boiler difficulties are met with, more especially in regard to nuisance from the chimney. This no doubt can be to a considerable extent avoided by introducing only a small quantity of the refuse at a time, but to do this involves a considerable addition to the work of the fireman, and keeps him tied to the furnace when he ought to be at other work, while it involves also a certain amount of delay in the destruction of the rubbish, which is a great evil, especially when it is kept in a warm place like the stoke-hole.

We recently inspected the destructor which has been erected at Guy's Hospital under the direction of Mr. Thomas Kirkland, M.Inst.C.E., of 17, Victoria Street, S.W., consulting engineer to the hospital, and as we are assured by Dr. Perry, the superintendent of the hospital, that it has now been in use for three years, and has given every satisfaction, we think a description of it will be of interest to our readers.

It must first, however, be noted that the problem is somewhat different from that which arises in devising a destructor for town refuse, where the action is continuous and the amount to be dealt with is very large. In this case the total amount is not very great, but sufficient of itself to produce all the heat required for its complete destruction, and the great mass of it comes down in the morning, and requires to be destroyed immediately. The apparatus, then, must be large enough to receive all that is likely to be brought down, it must be capable of producing heat enough thoroughly to burn all the gases evolved, and to completely incinerate the residue, and arrangements should be made for utilising under the boiler the heat produced by the combustion both of the waste material and of the coal used in the preliminary heating, and when the supply of refuse is exhausted.

As is shown by the accompanying section, the combustion takes place upon fire-bars under a brick arch, and it is thus possible to expose the resulting gases to a very high temperature. The refuse, however, does not fall at first upon the grate bars, but upon a flat, cast-iron "dead-plate"—where it lies until it is sufficiently roasted—the gases produced during this process passing over the hottest part of the fire and being thus effectually burned up.

The process then is this: The refuse is brought from the different parts of the hospital in iron barrows, which are wheeled up an incline to a steel floor, forming the top of the destructor, on which it is tipped. In this floor are two doors, and, one or other of them being opened, the refuse is raked in, and falls upon the dead-plate, and fills up the receptacle above it.

While it lies there the portion exposed to the heat of the furnace becomes dried, and the fumes evolved pass over the furnace and are consumed. After a time the furnace door is opened, and the desiccated mass is pushed on to the furnace bars, where its combustion is completed, and the residue becomes turned into clinker.

To prevent the bars becoming choked with this, they are capable of being moved by a rocking bar worked from the ash-door. The whole of the flames and gases impinge upon a fire-brick "bridge" just beyond the further end of the grate-bars, which becomes intensely hot, and thus their complete combustion is ensured. They then enter the internal flue of a boiler from which the ordinary furnace has been removed, and produce steam for engine and other purposes.

Just beyond the "bridge" there is chamber for the destruction of bedding. This has a separate door, by which an entire bed can be introduced without folding or any further manipulation than is required to push it in.

By passing the air through a cavity in the furnace walls it is to some extent warmed before arriving at the grate-bars, and thus heat is economised.

It is found in practice that the whole of the refuse of the institution is consumed in the course of the morning, and that during that time the heat from the destructor, which is entirely derived from the refuse burned, is sufficient to keep steam at thirty pounds per square inch in the boiler under which it is used, and there seems to be no doubt that a considerable economy is the result, as well as the complete carrying out of the primary object of the arrangement, viz., the destruction of the refuse. We were also assured that no nuisance arises from the chimney.

## Figures and Tables

**Figure f1:**